# Utilizing Olive Fly Ecology Towards Sustainable Pest Management

**DOI:** 10.3390/biology14020125

**Published:** 2025-01-25

**Authors:** Giorgos Stavrianakis, Efstratios Sentas, Sofia Zafeirelli, Thomas Tscheulin, Thanasis Kizos

**Affiliations:** 1 Rural Geography & Precision Farming Systems Lab, Department of Geography, University of the Aegean, Mytilene 81100, Greece; g.stavrianakis@aegean.gr (G.S.); s.sentas@aegean.gr (E.S.); s.zafeirelli@aegean.gr (S.Z.); 2 Biogeography & Ecology Lab, Department of Geography, University of the Aegean, Mytilene 81100, Greece; t.tscheulin@aegean.gr

**Keywords:** agroecology, olive fly, pest management, sustainability, *Bactrocera oleae*, climate change, sustainable agriculture, biological control

## Abstract

The olive fly (*Bactrocera oleae*, OLF) is a major pest of global significance that occurs in places where olive cultivation thrives. This paper highlights the economic and environmental damage caused by OLF infestations, including reduced olive oil yield and quality, disrupted supply chains, and ecosystem imbalances due to heavy insecticide use. Understanding olive fly ecology is crucial for developing effective control strategies. The review explores the fly’s life cycle, its relationship with olive trees, and how environmental factors like temperature and humidity influence population dynamics. Additionally, studying the role of natural enemies and agricultural practices can pave the way for sustainable control methods that minimize environmental harm. Climate change, intensive cultivation, and the development of resistance to insecticides necessitate a shift towards sustainable practices. This includes exploring alternative control methods like biological control with natural enemies and attract-and-kill strategies. Furthermore, a deeper understanding of OLF ecology, including its response to temperature and its ability to find refuge in diverse landscapes, is critical for predicting outbreaks and implementing effective protection strategies. By employing a holistic approach that integrates ecological knowledge with sustainable control methods, we can ensure the continued viability of olive cultivation, protect the environment, and produce high-quality olive oil.

## Simple Summary

The olive fly is a major pest that threatens olive trees worldwide. It attacks olives, reducing both the quantity and quality of the harvest. This can disrupt supply chains and lead to economic losses for farmers and businesses. To combat this pest, farmers often rely on chemical insecticides, which can harm the environment and beneficial insects. Understanding the olive fly’s life cycle and its relationship with olive trees is crucial for developing effective control strategies. Factors like temperature and humidity influence the fly’s population dynamics. By studying natural enemies and sustainable agricultural practices, we can explore alternative control methods that minimize environmental harm. Climate change and intensive cultivation have made olive fly a more serious threat. The development of insecticide resistance further complicates the problem. To address these challenges, we need to shift towards sustainable approaches. This includes using biological control agents like natural enemies and employing attract-and-kill strategies. Additionally, a deeper understanding of olive fly’s ecology, including its response to temperature and its ability to find refuge in different landscapes, is essential for predicting outbreaks and implementing effective protection measures. By combining ecological knowledge with sustainable control methods, we can protect olive trees, preserve the environment, and ensure the continued production of high-quality olive oil.

## 1. Introduction

The OLF, *Bactrocera oleae* (Rossi, 1790) (Diptera: Tephritidae), is one of the major pests of global significance to olive cultivation [1]. It is found almost anywhere cultivated olives are grown or wild olives naturally occur. The OLF has been reported in Africa, Europe, Canary Islands, Middle East, Chine, California, Mexico, and Central America [2]. The female lays its eggs directly within developing olive fruit, and the subsequent larval development causes significant economic losses. The economic losses caused by *B. oleae* worldwide are estimated to be around 800 million US dollars per year [3]. This makes *B. oleae* one of the most harmful olive pests globally [4]. As the larvae feed, they damage the fruit’s flesh, leading to premature ripening, oil quality degradation, and reduced fruit weight. Additionally, OLF infestations create suitable conditions for fungal pathogens, further compromising fruit quality and yield [5,6].

The economic impact of OLF infestations is substantial. Damaged olive fruits have lower market prices or become entirely unmarketable in the case of edible olives, while if they are intended for oil, its production, quality, composition and therefore price are affected. Large-scale outbreaks can cripple olive oil production in entire regions, impacting the livelihoods of farmers and disrupting global olive oil supply chains [7]. Beyond direct economic losses, OLF infestations can have significant ecological consequences. The extensive use of insecticides aimed at controlling OLF populations can disrupt natural ecosystems and harm beneficial insects like pollinators, predators, and parasitoids [8].

Estimates suggest that the OLF causes losses of 5% to 15% in the total olive production annually [2,9]. For example, in 2013, more than 3.6 million tons of olives and 3.27 million tons of olive oil were lost [6]. This damage manifests in several ways: the larvae consume the fruit pulp, leading to premature fruit drop, reduced oil yield, and compromised oil quality due to the increased acidity from microbial growth [10]. In severe infestations, the losses can reach up to 80% of the oil’s value and cause complete destruction of certain table cultivars [6,10]. The level of infestation is affected by many factors, such as the region, the climatic conditions, the tree varieties, and farming practices [11,12,13]. The pest’s ability to thrive in diverse environments further complicates management efforts, as it can adapt to various climatic conditions [7,14].

This review aims to provide a comprehensive overview of OLF ecology. By understanding how temperature, humidity, and other environmental factors influence OLF behavior, development, and population dynamics, particularly in the face of climate change, we can develop more sustainable and effective OLF management strategies. By mitigating the economic and ecological impact of this persistent pest, we can ensure the continued viability and sustainability of olive cultivation.

## 2. Biogeographic Patterns and General Biology

**Figure 1:**
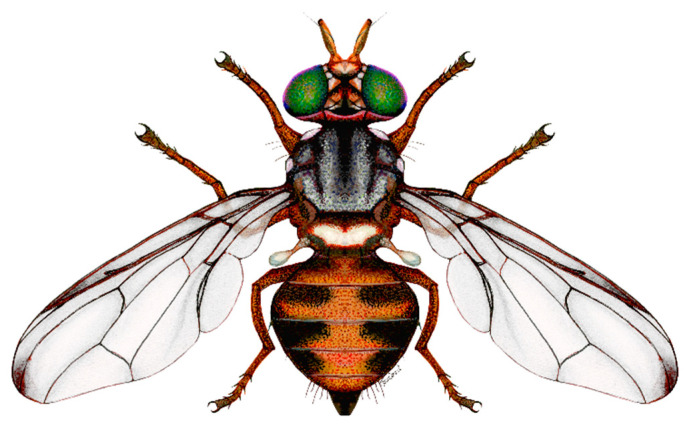
Olive fly, female adult.

An adult OLF is 4–5 mm long and ranges from light to dark brown. It has a whitish to yellowish scutellum, and the female has a prominent ovipositor (Figure 1). Its eggs are whitish and 0.7 mm long. The larva is whitish to yellowish when it grows in green fruits and purplish if found in dark, ripe fruits, reaching 7–8 mm. The pupa is ellipsoid and light brown [1].

During the OLF’s life cycle, it undergoes complete metamorphosis, transitioning through distinct egg, larval, pupal, and adult stages. Each stage plays a vital role in ensuring the fly’s survival and reproductive success. The OLF’s reproductive behavior is intricately linked to olive fruit development [15]. The mating process involves several distinct phases, including the search for mates, where males are attracted to females; courtship; and successful copulation [16]. Male OLFs engage in wing fanning behavior during courtship, which has been shown to increase their mating success [17]. Once mated, females meticulously select oviposition sites, preferring developing olive fruits with the optimal moisture content and minimal physical damage. Fecundity varies depending on the size of the female and environmental conditions, with a single female capable of laying 12 eggs a day and between 200 and 500 eggs throughout its lifespan [18,19].

Molecular techniques, such as chromosome quotient (CQ) analysis and mitochondrial genome sequencing, have been used to elucidate the taxonomic classification and evolutionary relationships of *B. oleae* and other *Bactrocera* species [20]. A molecular clock analysis based on a combination of cytochrome oxidase I (COI) and NADH dehydrogenase subunit 1 (NADH) gene sequences was used to resolve the taxonomic status of several *Bactrocera* species. The analysis showed that *B. oleae* diverged from other *Bactrocera* species, such as *B. papayae* and *B. carambolae*, around 2.7 million years ago [21]. The OLF has a widespread distribution, primarily in the Mediterranean Basin, but also in other regions where olive cultivation is present. The native range of the insect is centered in the Afrotropics, including parts of Africa, Asia, and the Mediterranean region [22]. However, the pest has also been introduced and established in other areas, such as North and Central America, where it has become a significant problem for olive growers [23]. The distribution and spread of OLF are influenced by various factors, including climate, landscape structure, and the availability of its host plant, the olive tree (*Olea europaea* L.) [3,24].

**Figure 2:**
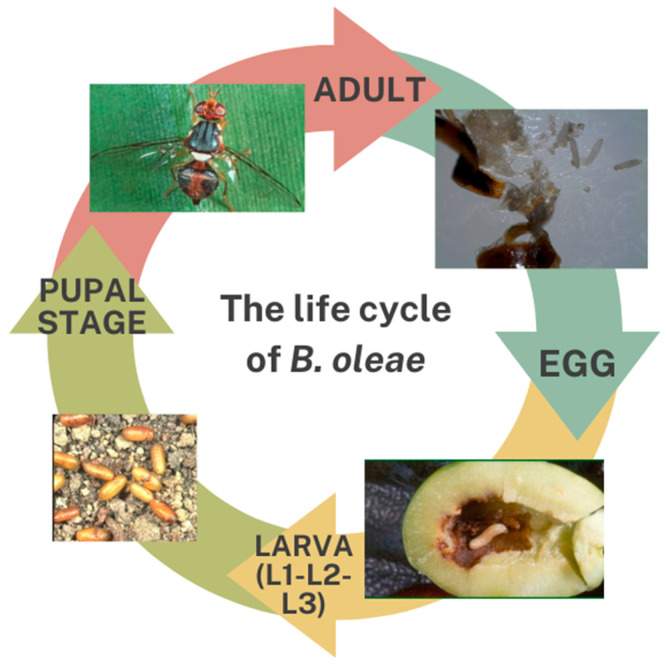
The life cycle of *B. oleae*.

Its development and survival rates differ across each life stage (Figure 2). Under the optimal conditions (10 °C−30 °C), its eggs hatch in 1 day, giving rise to larvae that are vulnerable to predators [6]. These larvae primarily feed on the olive fruit’s pulp, with their development influenced by temperature and fruit quality. Warmer temperatures can accelerate larval development, while unfavorable temperatures (<23 °C, >29 °C) or damaged fruit can lead to increased mortality [25,26]. Following three instars (molting stages), the larvae open an exit hole in the olive epicarp and either escape from the fruit to pupate in the soil or pupates inside the fruit and open an exit hole for the adult [6]. Studies have found that larvae pupates in the top 3 cm [27], while other research studies have found that they can reach the depth of 7 cm in the soil [28]. The pupal stage lasts for several weeks, with the emergence timing depending on temperature [29]. Adult flies that emerge from pupae will seek out mates and suitable oviposition sites, perpetuating the cycle up to 6–7 generations per year. A comprehensive understanding of the factors influencing each life stage of the fly is paramount to developing targeted OLF management strategies that can disrupt specific phases of its development and reproduction.

Pupation occurs within olive fruits during the summer season, while during autumn, the majority of the third instar larvae exit the olive to pupate and undergo overwintering in the soil. Additionally, in regions characterized by mild winter climates, the OLF can survive the winter either as larvae or adults [30,31]. In the ensuing spring months, adults emerge between March and May, initiating a series of generations ranging from one to five, contingent upon environmental factors [31]. The OLF can complete a generation in as little as 30 to 35 days at optimum temperatures. Optimal temperatures ranging from 22 °C to 26 °C promote higher infestation rates [32,33].

The female introduces an egg into the pulp (mesocarp) of green or ripe olive fruits after having made an oviposition slit in the epicarp using her ovipositor and a minute chamber underneath in the pulp [1]. In mid-summer, females start to lay eggs (1 egg per olive—except in cases of very dense populations—until 12 eggs/day) and continue until late autumn, while each one can lay between 200 and 500 eggs [34,35].

## 3. Host Plants and Interactions

The OLF exhibits a strong association with its primary host plant, the *Olea* species (mostly the widespread *Olea europaea* L.), but its interactions with other plant species and microorganisms are also significant [10,36]. Olive fruits serve as the sole breeding ground for the fly, providing sustenance for developing larvae and ensuring the continuation of the OLF’s lineage [2]. The OLF has developed a symbiotic relationship with the bacterium *Candidatus* Erwinia dacicola, which aids in overcoming the plant’s chemical defenses, particularly oleuropein, a bitter compound found in olives [37,38]. This symbiosis enhances the fly’s ability to exploit olive fruits, thereby increasing its reproductive success. Moreover, the OLF’s attraction to specific volatile compounds produced by both the olive fruit and associated microorganisms has been documented [39,40]. These interactions underscore the complexity of the OLF’s ecological niche and its reliance on both plant and microbial partners for survival and reproduction.

The larvae’s ability to thrive in unripe fruits, which possess strong antimicrobial properties, is a unique adaptation that allows them to exploit a niche that is often less competitive [41,42]. The OLF’s reproductive success is influenced by various factors, including the physical characteristics of the fruit. Research has shown that the fruit’s texture can affect oviposition preferences, with softer fruits being more susceptible to infestation [43]. Furthermore, the presence of specific volatiles emitted by host plants plays a crucial role in attracting adult flies for mating and oviposition [44,45]. The olive tree’s unique chemical profile, including the volatile organic compounds emitted during fruit development, acts as a powerful attractant for adult olive flies searching for oviposition sites. Additionally, the olive fruit’s fleshy mesocarp offers a perfect nutritional source for larvae, facilitating their growth and development [46].

While the olive tree stands as the OLF’s primary host, the potential role of alternative host plants in OLF population dynamics cannot be ignored. Certain wild olive relatives and other fruiting trees have been documented to harbor OLF populations [47]. Adult OLFs, during the search for their host plant, can use a variety of food sources, different pollens, honeydews, fruit and plant exudates, and bacteria [31]. They have been found on plant species such as lemon, cypress, fig, and orange trees [48,49,50]. While some studies suggest these alternative hosts may serve as reservoirs for OLF populations, particularly during periods of low olive fruit availability, their overall contribution to OLF population dynamics remains a subject of ongoing research [51].

The OLF’s interaction with the olive tree is a complex interplay of attraction, exploitation, and potential harm [52]. As mentioned earlier, the olive’s volatile compounds lure adult flies, guiding them towards suitable oviposition sites [44,53], specifically toluene, C6 compounds from the lipoxygenase pathway, hexanal, (E)-2-nonenal, and specific sesquiterpenes [44,54]. The subsequent egg-laying and larval development within the olive fruit translates into a significant investment of resources by the tree [23,55]. The OLF’s feeding activity damages the fruit pulp, leading to premature ripening, oil quality degradation, and reduced yield [9,13,56,57,58,59]. Additionally, the fly may introduce pathogens with its feeding, further compromising fruit health [60,61,62,63,64]. Understanding the factors influencing host selection and the impact of OLF infestation on fruit quality is crucial for developing effective control strategies that protect olive trees and ensure sustainable olive production [52].

## 4. Environmental Factors

**Figure 3:**
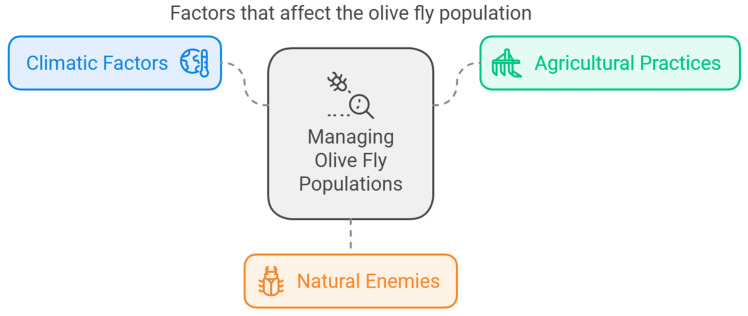
Factors that affect the olive fly population.

The OLF’s population dynamics, development, and activity are intricately interwoven with climatic factors, and the presence of native aromatic species, such as *Cistus creticus* L. and *Lavandula stoechas* L., in understory plant communities can either promote or inhibit OLF infestations [32,65] (Figure 3). Temperature plays a pivotal role, influencing egg development, larval growth rates, and adult emergence times [66]. Warmer temperatures can accelerate OLF development, leading to faster population turnover and potentially higher infestation levels [67]. Conversely, cool temperatures can slow development and reduce overall population growth. The optimal temperature range, according to the research, is 23–29 °C [6]. Rainfall and humidity also have a significant influence, with the optimal level of relative humidity according to the research being around 60% (RH) [68]. While some level of humidity is necessary for egg development and adult survival, excessive rainfall can disrupt mating behavior and decrease egg viability. Humidity fluctuations can similarly impact development rates and adult activity levels. Understanding these climatic interactions is crucial for predicting OLF outbreaks and implementing targeted control measures. Furthermore, climate change poses a potential threat to olive cultivation and OLF management strategies. Predictive models indicate that shifts in climate could alter the geographic distribution and abundance of OLF populations towards northern regions because of the changes in the suitability of different regions for olive cultivation [3,7,66], complicating existing management practices [7]. Understanding these environmental interactions is crucial for developing effective control strategies for more precise and low-input practices.

Agricultural practices also play a part in shaping OLF populations. Irrigation practices can influence fruit quality and susceptibility to attack. For instance, water stress in olive trees may lead to increased fruit sugar content, making them more attractive to egg-laying females. Conversely, fertilization practices can indirectly negatively affect OLF populations by influencing the abundance and diversity of natural enemies within the olive grove ecosystem [69,70].

Natural enemies, encompassing predators and parasitoids, play a vital role in regulating OLF populations. Predatory insects like ladybugs and ground beetles feed on OLF eggs and larvae, spiders, birds, bats, and lizards predate on OLF adults, while parasitoid wasps lay their eggs within OLF pupae, effectively reducing fly emergence rates [30,71]. Maintaining a healthy and diverse population of these natural enemies through the use of organic farming practices and the creation of a suitable habitat within olive groves offers a sustainable approach to OLF control [30,72]. By understanding the complex interplay between climatic factors, agricultural practices, and natural enemies, we can develop more sustainable and holistic strategies for managing OLF populations and ensuring healthy olive production.

## 5. Management Strategies

**Figure 4:**
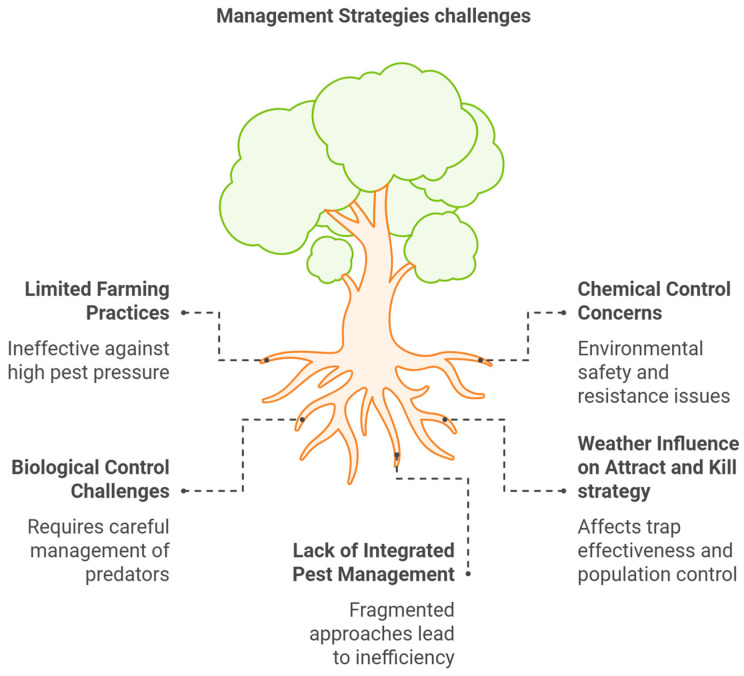
Management strategy challenges.

The OLF presents a persistent challenge for olive growers (Figure 4). Diverse methods and control strategies have been developed to combat this pest [73]. Farming practices represent the first line of defense [72,74,75]. Early harvesting, before peak OLF activity, can significantly reduce fruit damage [76,77]. Sanitation measures, such as removing fallen fruit and pruning to improve orchard ventilation, also play a role by minimizing potential breeding sites [57,78]. However, the effectiveness of farming practices alone is often limited, particularly in areas with high OLF pressure [2,79,80].

Chemical control, through the application of insecticides, has traditionally been the mainstay of OLF management [81]. Spraying applications are carried out using two methods: (i) bait sprays and (ii) cover sprays. Bite sprays use insecticides, such as organophosphate (at a concentration of 0.3% active ingredients), combined with a food attractant, such as 2% hydrolysate protein. This mixture is then applied from ground level to a limited section of the canopy, typically ranging from 1 to 2 cubic meters. The amount of spraying solution per tree does not exceed 300 milliliters. Cover sprays involve using insecticide at a concentration that is ten times lower (e.g., 0.03% organophosphate) compared to that in bait sprays. Moreover, cover sprays require a significantly larger volume of spray solution (approximately 10 to 15 L per tree) to be applied across the entire tree canopy until runoff [82].

However, concerns over environmental safety, insecticide resistance development in OLF populations, and potential negative impacts on beneficial insects have spurred the exploration of alternative methods [83].

Biological control offers a more sustainable approach by harnessing the power of natural enemies. The release of predatory insects or the promotion of existing predator populations within an olive grove can help regulate OLF numbers [84,85]. Additionally, the use of parasitoid wasps that target OLF pupae is gaining traction. While promising, biological control often requires careful management to establish and maintain effective predator and parasitoid populations [86]. This can be achieved by maintaining native flora inside and at the edges of the olive groves [72]. Moreover, it has been proven that certain herbs, such as *Cistus creticus* L. and *Lavandula stoechas* L., can act as repellents for the OLF because of their olfactory cues, which can reduce fly attraction to odors from the olive host by 10% [65]. Additionally, the identification of beneficial soil arthropods that can naturally suppress OLF populations has opened new avenues for integrated pest management strategies [71]. Recent studies have also focused on the role of microbial antagonists in controlling OLF populations. The use of bacterial isolates, such as *Providencia entomophila*, has been shown to increase the mortality rates in olive flies, suggesting that these microorganisms could serve as biocontrol agents [87].

The development of biopesticides based on these natural enemies could provide a sustainable solution to OLF management, reducing the need for synthetic chemicals. Entomopathogenic fungi, such as *Metarhizium brunneum* and *Beauveria bassiana*, have shown efficacy in controlling the OLF and other Tephritid fruit fly pests [88,89,90,91,92]. These fungi can infect and kill the pest through direct contact, and some can also have sublethal effects on the pest’s behavior and reproduction [93]. *Metarhizium brunneum* can be used for soil-based treatments targeting the pre-imaginal stages of the OLF [88,89]. This approach has been shown to reduce the emergence of adult flies by significant percentages in treated plots compared to controls [89]. The persistence and efficacy of these fungal treatments can be influenced by factors such as the formulation type and environmental conditions [88].

Additionally, entomopathogenic fungi can be integrated with other management strategies, such as the use of pheromone-based attractants [45] and the development of molecular tools for pest detection and monitoring [56,94], to create a more comprehensive and effective integrated pest management approach against the OLF [2]. Interactions between entomopathogenic fungi, plants, and insects can be complex, and plant-mediated effects may influence the efficacy of these fungi as biocontrol agents [95]. Understanding these interactions could help optimize the use of entomopathogenic fungi in olive production systems.

Attract-and-kill (A&K) strategies utilize pheromone and food attractant traps that lure adult olive flies and trap them, effectively reducing mating success and egg-laying. These approaches are environmentally friendly and require less broad-spectrum insecticide application. However, their effectiveness can be influenced by weather conditions and OLF population densities [35].

One of the most promising technological advances in OLF management is the application of a sterile insect technique (SIT). This method involves the mass rearing of sterile male flies, which are then released into the wild to mate with wild females, resulting in no offspring and a gradual reduction in the population. Recent studies have demonstrated the feasibility of using genetically enhanced sterile insect techniques, such as the fsRIDL (female-specific Release of Insects carrying a Dominant Lethal) approach, which has shown promise in suppressing OLF populations in controlled environments [34]. This method not only reduces the reliance on chemical insecticides but also minimizes the ecological impact associated with traditional pest control methods. Furthermore, advancements in the understanding of the OLF’s genetic makeup have facilitated the identification of specific traits that can be targeted for control. For instance, research into the gut microbiota of *Bactrocera oleae* has revealed potential avenues for manipulating these microbial communities to enhance pest management strategies [96,97]. By understanding the symbiotic relationships between the OLF and its gut bacteria, researchers can develop targeted interventions that disrupt these interactions, potentially leading to reduced pest viability.

Technological advancements in monitoring systems have significantly improved the ability to track OLF populations and inform management decisions. The development of decision support systems (DSSs) that integrate location-aware technologies allows farmers to receive real-time data on pest populations and environmental conditions [98,99,100]. These systems can provide tailored recommendations for pest management, optimizing the timing and application of control measures. Moreover, the use of attract-and-kill devices, which combine attractants with insecticides, has emerged as a promising strategy for managing OLF populations. These devices minimize the impact of full-cover spraying and target specific pest populations, thereby reducing the overall chemical load in the environment [85]. Research into new attractants derived from yeast associated with the olive fruit fly has further enhanced the effectiveness of these devices [45]. While the attract-and-kill approach is targeted towards OLF, there is a risk of non-target effects on other arthropod species in the olive grove ecosystem [31,101]. The use of insecticides, even in targeted bait stations, can still pose risks to other arthropods, including pollinators and natural enemies of OLF [31,102].

The future of OLF management lies in the development and implementation of integrated pest management (IPM) programs [103]. These programs combine various control strategies, including cultural practices, biological control, A&K techniques, and the judicious use of selective insecticides, to achieve long-term, sustainable OLF suppression [104]. This holistic approach minimizes the environmental impact while effectively managing OLF populations and ensuring high-quality olive production [103]. As part of an IPM program, farmers could opt to cultivate olive cultivars that are more resistant to OLF infestation. The susceptibility of olive cultivars to the OLF may be influenced by various factors, including the physical and chemical characteristics of the olive fruit, such as epicuticular waxes [105] and volatile organic compounds [44]. These cultivar-specific traits can affect the olive fly’s ability to detect, access, and oviposit on the fruit. Additionally, there is genetic variation in olives’ resistance to the OLF, so the identification and use of resistant cultivars could be an important tool for IPM programs [12,106]. However, “resistant” olive cultivars are not completely immune to OLF infestation [9]. The pest can still cause significant damage to these varieties, though potentially at lower levels compared to that in more susceptible cultivars. Moreover, we should not underestimate the importance of local varieties and the importance of their conservation.

## 6. Technological Advances

Recent technological advances have substantially enhanced OLF population monitoring and pest control strategies. Miranda et al. developed a decision support system (DSS) for managing the olive fruit fly in Mediterranean olive orchards, utilizing semi-automatic, location-aware, real-time monitoring that significantly enhanced the pest control efficiency [98]. They integrated electronic traps that monitored pest populations with georeferenced tree data, enabling precise, targeted insecticide applications. Based on such frameworks, further innovations have introduced electronic traps (e-traps). An example is the research by Diller et al., where deep learning algorithms are used to monitor fruit fly populations while enabling real-time data acquisition and analysis [107].

Further technological advances in OLF population monitoring and intervention have been achieved through machine learning techniques and deep learning techniques, aiming at the detection and counting of olive fruit flies. The study in Molina-Rotger et al. investigated the potential of machine learning (ML) techniques in electronic traps for remote monitoring of pests [108]. They used Random Forest (RF) and Support Vector Machine (SVM) algorithms to develop a tracking system, achieving a significant detection accuracy, enabling the remote tracking and detection of pests to facilitate precision agriculture. This approach reduces the need for on-site monitoring of olive groves and supports sustainable pest management by optimizing pesticide use and minimizing the ecological impact. In Mamdouh et al., an attempt is made to detect olive fruit flies captured in smart traps, proposing a specialized framework that exploits the strengths of the YOLO deep learning algorithm, adapted to olive fruit fly recognition and counting [109]. The algorithm is trained from images of smart pheromone traps. Yellow color normalization is applied, where the images are adjusted to the yellow color of the traps, making the framework illumination-invariant. For greater accuracy, the training dataset is enriched with negative samples, as well as data augmentation techniques being applied.

In addition to assessing the accuracy of OLF detection algorithms, Mira et al. also took the energy consumption into account [110]. They used three computer vision methods for object classification to detect the OLF in colored traps. The images used to train the algorithm consisted of a variety of realistic conditions to maximize the accuracy.

## 7. Discussion

The relentless quest for effective OLF management necessitates continuous research and exploration. Several key areas demand further investigation to ensure the sustainability of olive production in the face of evolving challenges. A crucial area of research concerns the development of resistance in OLF populations to the existing control methods. The widespread use of insecticides has been selected for resistant fly strains, jeopardizing the effectiveness of chemical control programs [2,111,112]. For example, in Greece, there are reports of resistance to pyrethroid and alpha-cypermethrin insecticides [113,114]. Future research should focus on monitoring and understanding the evolution of their resistance mechanisms, while simultaneously exploring alternative insecticides with novel modes of action that can circumvent the existing resistance [113,115].

The looming threat of climate change necessitates a deeper understanding of its impact on OLF ecology [46,66]. Rising temperatures and altered precipitation patterns are predicted to influence OLF population dynamics, development rates, and geographical distribution. Research efforts should focus on simulating climate change scenarios and their potential consequences for OLF populations [106,116,117]. By understanding how climate change will reshape the OLF’s behavior and development, we can develop proactive management strategies that remain effective under future climatic conditions [7,118,119].

The use of an integrated geoinformatics system for OLF monitoring and spraying could be an important factor for timely and effective control of this insect [120]. Exploiting the possibility of mapping the trap network and recording real-time data on the OLF population enable the direct identification of outbreaks and the correlation of insect density with various environmental parameters [98]. By tracking the spray vehicles, it is possible to monitor in real time the areas where spraying is carried out and to take corrective measures [121]. The use of the above applications leads to better management of insecticides and therefore has a positive environmental impact [122].

Finally, exploring novel control strategies holds immense promise for sustainable OLF management. Research efforts should investigate the potential of utilizing semiochemicals, such as kairomone lures that attract natural enemies, or the development of genetically modified olive trees with increased resistance to OLF infestation [44]. Additionally, advancements in biocontrol techniques, such as the mass rearing and release of effective parasitoid wasp species, need further exploration [123,124,125]. By investing in these innovative approaches, we can create a diverse toolbox for OLF control, ensuring the long-term viability and environmental sustainability of olive production.

The olive fly is one of the major threats to olive production, causing significant economic and environmental damage [3,80,126]. Infestations reduce olive oil yield and quality, impacting farmers’ income and disrupting global supply chains [6]. Additionally, the heavy use of insecticides to control these pests harms beneficial insects and disrupts ecosystems [127,128].

To effectively manage this persistent pest, scientists need a deep understanding of OLF ecology. This review delves into the fly’s life cycle, its relationship with its primary host (the olive tree), and how environmental factors like temperature and rainfall influence its population dynamics [24,129]. By understanding these aspects, researchers can develop targeted control strategies [130]. For instance, studying each life stage’s vulnerabilities allows for interventions that disrupt specific parts of the fly’s development and reproduction. Additionally, exploring the role of natural enemies and the impact of agricultural practices can lead to the development of sustainable control methods that minimize environmental harm [80].

Research shows that to effectively control the OLF, all of the parameters related to its ecology must be taken into account. It is clear that climate change, intensive cultivation, environmental degradation, and the development of resistance to agrochemicals, combined with the existence of alternative hosts and the insect’s ability to cover considerable distances during the day, are creating new data for understanding and effectively managing it [100]. Research shows that the insect can take refuge in nearby areas when conditions are unfavorable for it [30]. In many cases, we cannot talk about population reductions under extreme weather conditions but about the population finding shelter in a more diverse landscape so that it can return when these conditions are favorable. An understanding of these parameters, further study of the ecology of the OLF, and a more environmentally friendly approach to the subject could lead to the prediction of population outbreaks and more effective protection of olive groves [100].

Compared to other Tephritid fruit flies, such as *Ceratitis capitata*, while the management strategies share some similarities, such as the use of biological control and IPM approaches, the specific challenges and solutions may vary due to the unique biology and ecology of each pest species. The development of insecticide resistance is a common challenge across Tephritid fruit fly pests, highlighting the need for alternative, sustainable control methods [2,50]. The role of the gut microbiome in the biology and management of Tephritid fruit flies, including *B. oleae*, is an emerging area of research that may lead to novel control strategies [50,80]. Meanwhile, molecular techniques, such as DNA barcoding and PCR-based diagnostics, have proven valuable for the identification and monitoring of Tephritid fruit fly species, which can support the implementation of effective management programs [94,131].

## 8. Conclusions

Despite the significant economic impact of the OLF on olive production worldwide, our understanding of its ecology remains incomplete. This gap in the knowledge presents a critical barrier to developing truly effective, precise, and sustainable management strategies. Further research is needed to elucidate key aspects of the OLF’s life cycle, behavior, and interactions with the environment. This includes investigations into the factors influencing population dynamics, host–parasite interactions, and the impact of climate change on the OLF’s distribution and abundance. We need to treat the OLF not only as a pest but firstly as an insect as a part of the agroecosystem. By filling these knowledge gaps, we can refine the existing management practices, develop novel control methods, and ultimately minimize the negative impacts of the OLF on olive production while preserving environmental health.

Successful OLF control offers significant economic and environmental benefits. Reduced crop losses translate into higher olive oil production and economic gains for farmers. Moreover, sustainable control methods minimize the reliance on harmful insecticides, promoting biodiversity and healthy ecosystems within olive groves. In conclusion, a comprehensive understanding of OLF ecology is the key to developing effective and sustainable management strategies, ensuring the continued viability of olive cultivation and the production of high-quality olive oil.

## Data Availability

Not applicable.
